# *Astragalus membranaceus* Extract Induces Apoptosis via Generation of Reactive Oxygen Species and Inhibition of Heat Shock Protein 27 and Androgen Receptor in Prostate Cancers

**DOI:** 10.3390/ijms25052799

**Published:** 2024-02-28

**Authors:** Seok-Young Kim, Ji Eon Park, Hyo-Jung Lee, Deok Yong Sim, Chi-Hoon Ahn, Su-Yeon Park, Bum-Sang Shim, Bonglee Kim, Dae Young Lee, Sung-Hoon Kim

**Affiliations:** 1 College of Korean Medicine, Kyung Hee University, Seoul 02447, Republic of Korea; amabledaniel@naver.com (S.-Y.K.); wdnk77@naver.com (J.E.P.); hyonice77@naver.com (H.-J.L.); simdy0821@naver.com (D.Y.S.); ach2565@naver.com (C.-H.A.); waterlilypark@naver.com (S.-Y.P.); eshimbs@khu.ac.kr (B.-S.S.); bongleekim@khu.ac.kr (B.K.); 2 Department of Herbal Crop Research, National Institute of Horticultural and Herbal Science, Rural Development Administration, Eumseong 27709, Republic of Korea; dylee0809@gmail.com

**Keywords:** apoptosis, AR, *Astragalus membranaceus*, HSP27, prostate cancer, ROS

## Abstract

Although *Astragalus membranaceus* is known to have anti-inflammatory, anti-obesity, and anti-oxidant properties, the underlying apoptotic mechanism of *Astragalus membranaceus* extract has never been elucidated in prostate cancer. In this paper, the apoptotic mechanism of a water extract from the dried root of *Astragalus membranaceus* (WAM) was investigated in prostate cancer cells in association with heat shock protein 27 (HSP27)/androgen receptor (AR) signaling. WAM increased cytotoxicity and the sub-G1 population, cleaved poly (ADP-ribose) polymerase (PARP) and cysteine aspartyl-specific protease 3 (caspase 3), and attenuated the expression of B-cell lymphoma 2 (Bcl-2) in LNCaP cells after 24 h of exposure. Consistently, WAM significantly increased the number of terminal deoxynucleotidyl transferase dUTP nick end labeling (TUNEL)-positive LNCaP cells. WAM decreased the phosphorylation of HSP27 on Ser82 and inhibited the expression of the AR and prostate-specific antigen (PSA), along with reducing the nuclear translocation of p-HSP27 and the AR via the disturbed binding of p-HSP27 with the AR in LNCaP cells. WAM consistently inhibited the expression of the AR and PSA in dihydrotestosterone (DHT)-treated LNCaP cells. WAM also suppressed AR stability, both in the presence and absence of cycloheximide, in LNCaP cells. Taken together, these findings provide evidence that WAM induces apoptosis via the inhibition of HSP27/AR signaling in prostate cancer cells and is a potent anticancer candidate for prostate cancer treatment.

## 1. Introduction

Androgens and androgen receptor (AR) signaling play important roles in the normal prostate and prostate cancer (PCa) development, maintenance, and progression, and as biomarkers for prostate cancer [1,2,3].

Recent research has revealed that HSP27 promotes nuclear trafficking and binding to the promoter region of the AR, thereby enhancing its transcriptional activity [4,5,6]. HSP27/AR signaling is closely associated with prostate cancer progression and resistance to androgen ablation [7]. Therefore, the regulation of HSP27 and the AR in prostate cancer cells is regarded as a good strategy for successful cancer therapy.

Recently, research was performed on natural compounds targeting the transcription and expression of the AR by inhibiting HSP27 [8]. Xanthone and flavonoid compositions (luteolin, quercetin, and kaempferol) inhibited the AR and its related gene products by inhibiting HSP27 [9,10].

*Astragalus membranaceus* (syn. *Astragalus propinquus*) [11], which has been traditionally used for immune enhancement and as a tonic in folk remedies for years, is known to have anti-obesity [12], anti-inflammatory [13], and anti-cancer effects [14]. Particularly, Astragalus polysaccharide, a bioactive component of dried *Astragalus membranaceus* roots [15], shows anti-proliferative, anti-metastatic, and apoptotic activity in gastric [16], breast [17], cervical [18], and lung [19] cancer cells. Nevertheless, the underlying molecular apoptotic mechanisms of *Astragalus membranaceus* are not fully understood in association with HSP27 and the AR in prostate cancer.

Hence, in the present study, the apoptotic mechanism of *Astragalus membranaceus* water extract (WAM) was elucidated in association with HSP27/AR signaling in prostate cancer cells. Here, we show the novelty of this manuscript, revealing that water extract of *Astragalus membranaceus* significantly exerts an apoptotic effect through the inhibition of the PSA and AR, specifically in AR-dependent LNCaP prostate cancer cells, not in AR-independent prostate cancers.

## 2. Results

### 2.1. WAM Reduced Viability and Increased PARP Cleavage in LNCaP Cells

**Figure 1:**
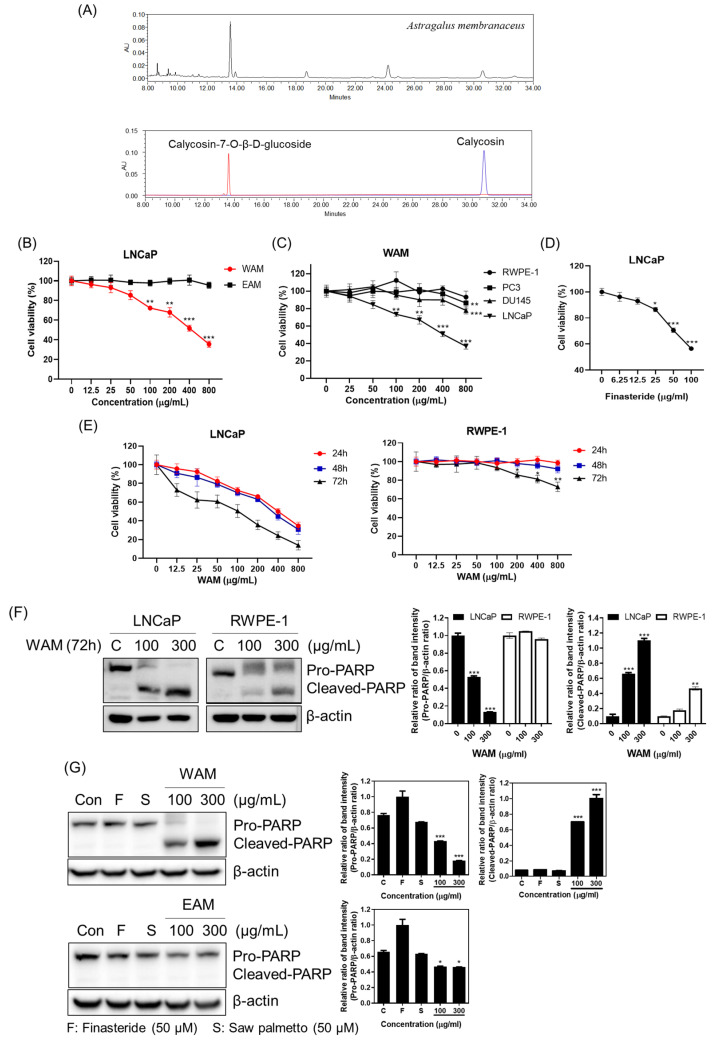
Cytotoxic effect of *Astragalus membranaceus* water extract in LNCaP prostate cancer cells according to HPLC analysis. (**A**) HPLC chromatogram of *Astragalus membranaceus* and overlay chromatogram of standard compounds. (**B**) Effect of WAM and EAM on the viability of LNCaP cells. LNCaP cells were treated with various concentrations of water extract from *Astragalus membranaceus* (WAM) or ethanol extract from *Astragalus membranaceus* (EAM) for 24 h, and cell viability was evaluated using an MTT assay. ** *p* < 0.01, *** *p* < 0.001 vs. untreated control. (**C**) Effect of WAM on the viability of RWPE-1, PC3, DU145, and LNCaP cells. Cells were treated for 24 h with various concentrations of WAM and subjected to MTT assay. ** *p* < 0.01, *** *p* < 0.001. (**D**) Effect of finasteride on the viability of LNCaP cells. LNCaP cells were treated with finasteride (50 μM) for 24 h and subjected to MTT assay. * *p* < 0.05, *** *p* < 0.001. (**E**) Effect of WAM on the viability of LNCaP and RWPE-1 cells. Cells were treated with various concentrations of WAM for 24 h, 48 h, and 72 h and subjected to MTT assay. (**F**) Effect of WAM on pro-PARP and cleaved PARP in LNCaP and RWPE-1 cells. Cells were treated with WAM (0, 100, 300 μg/mL) for 72 h and subjected to Western blotting. ** *p* < 0.01, *** *p* < 0.001. (**G**) Effect of WAM, finasteride, and saw palmetto on pro-PARP and cleaved PARP in LNCaP cells. LNCaP cells were exposed to WAM (0, 100, 300 μg/mL), finasteride (50 μM), and saw palmetto (50 μM), and were subjected to Western blotting. Expression levels of pro-PARP and cleaved PARP were evaluated in LNCaP cells. Data are expressed as means ± SD. * *p* < 0.05.

To confirm the leading compounds of *Astragalus membranaceus* extracts, HPLC analysis was conducted. As shown in Figure 1A, several compounds were revealed, including calycosin and calycosin-7-O-β-D-glucoside. To evaluate the cytotoxic effect of *Astragalus membranaceus*, a cell viability assay was performed in LNCaP human prostate cancer cells using an MTT assay. LNCaP cells were treated with the indicated concentrations (0, 12.5, 25, 50, 100, 200, 400, 800 μg/mL) of water extract from *Astragalus membranaceus* (WAM) and ethanol extract from *Astragalus membranaceus* (EAM) for 24 h. WAM suppressed the viability of LNCaP cells compared with EAM (Figure 1B). In addition, LNCaP cells were the most susceptible to WAM when compared with PC3, DU145, and RWPE1 cells (Figure 1C), though the efficacy of WAM was not comparable to finasteride (Figure 1D). Next, the time- (24, 48, and 72 h) and concentration-dependent effects of WAM were evaluated in LNCaP cells. Here, WAM exerted effective cytotoxicity in a time- and concentration-dependent manner (Figure 1E), with more significant cytotoxicity shown in LNCaP and RWPE-1 cells. Consistently, WAM significantly induced PARP cleavage, even in RWPE-1 cells, after 72 h of exposure (Figure 1F). It is noteworthy that WAM induced the cleavage of PARP in LNCaP cells, even after 24 h of exposure, compared to EAM and the untreated control (Figure 1G).

### 2.2. WAM Decreased AR and PSA Protein Levels in LNCaP Cells

**Figure 2:**
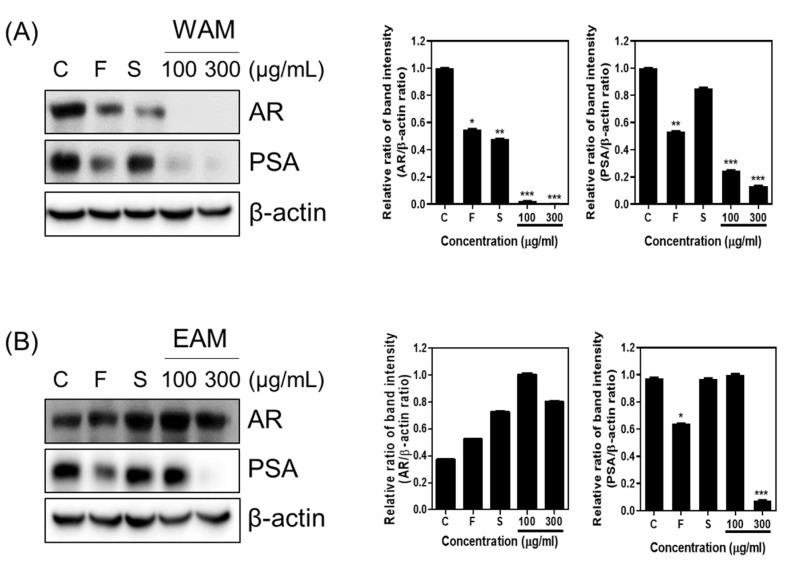
WAM suppressed the expression of the AR and PSA in LNCaP cells. LNCaP cells were treated with WAM or EAM (100, 300 μg/mL), finasteride (50 μM), and saw palmetto (50 μM). (**A**) Effect of WAM on AR and PSA protein expression in LNCaP cells. (**B**) Effect of EAM on AR and PSA protein expression in LNCaP cells. Data are expressed as means ± SD. * *p* < 0.05, ** *p* < 0.01, *** *p* < 0.001.

To determine the effect of WAM and EAM on the AR and PSA protein levels in LNCaP cells, the cells treated with various concentrations of WAM or EAM were subjected to Western blotting. WAM effectively reduced the expression of the AR and PSA in LNCaP cells (Figure 2A). In contrast, EAM decreased the expression of the PSA, while it did not affect the expression of the AR in LNCaP cells (Figure 2B).

### 2.3. WAM Induced Apoptosis in LNCaP Cells

**Figure 3:**
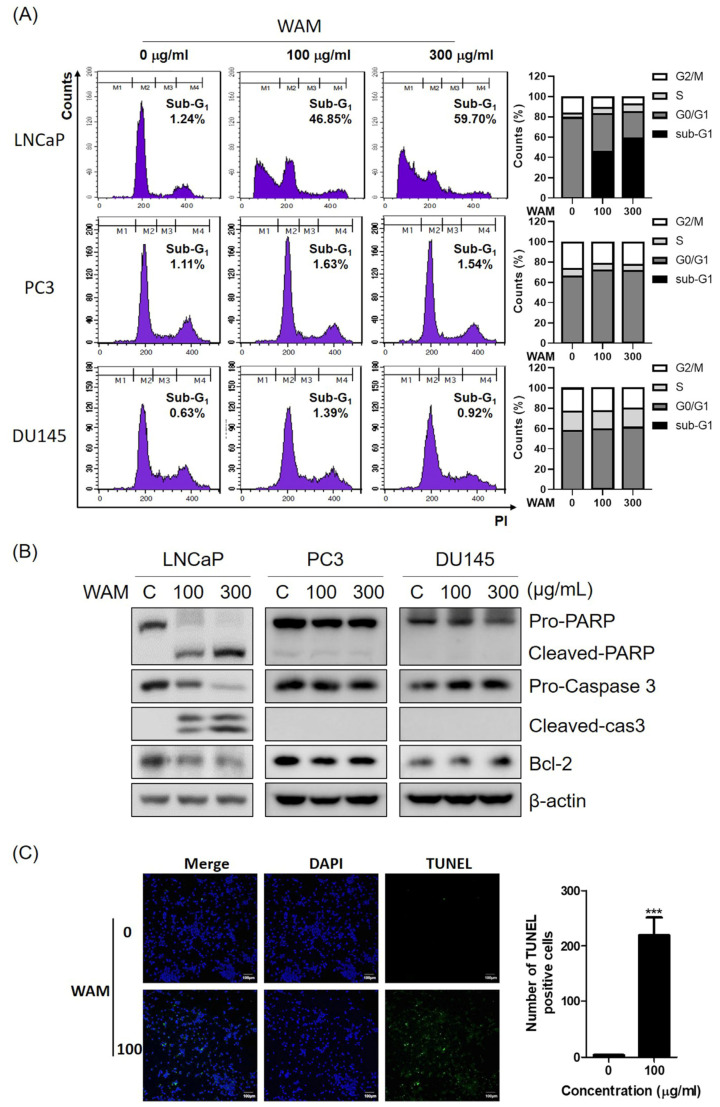
WAM induced apoptosis in LNCaP cells. (**A**) Effect of WAM on the sub-G1 population in LNCaP, PC3, and DU145 cells. The cells treated with WAM were stained with propidium iodide (PI) for cell cycle distribution with flow cytometry. (**B**) Effect of WAM on PARP, caspase 3, and Bcl-2 in LNCaP, PC3, and DU145 cells. The cells were exposed to WAM (100, 300 μg/mL) and were subjected to Western blotting with antibodies against PARP, caspase-3, cleaved caspase-3, Bcl-2, and β-actin. (**C**) Effect of WAM on TUNEL-positive cells in LNCaP cells. Scale bar, 100 μm. Data are expressed as means ± SD. *** *p* < 0.001.

To investigate the apoptotic effect of WAM, a cell cycle assay and Western blotting were performed in LNCaP, PC3, and DU145 cells. WAM increased the sub-G_1_ population in LNCaP cells (Figure 3A), but not in PC3 and DU145 cells. Consistently, WAM also increased the cleavage of PARP and caspase 3 and attenuated the expression of Bcl-2 in LNCaP cells, but not in PC3 and DU145 cells (Figure 3B). Thus, it was confirmed that WAM increased the number of TUNEL-positive cells in LNCaP cells compared to the untreated control (Figure 3C).

### 2.4. WAM Regulated Apoptosis through ROS Generation in LNCaP Cells

**Figure 4:**
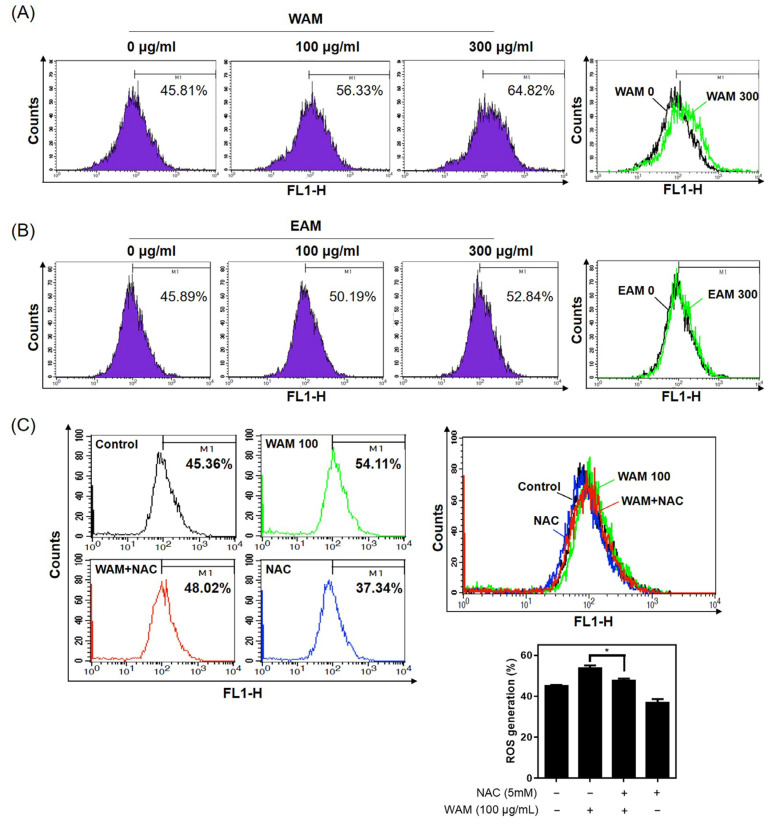
WAM increased ROS generation in LNCaP cells. LNCaP cells were treated with WAM (**A**) and EAM (**B**) for 24 h and subjected to ROS measurement using an oxidation-sensitive fluorescent dye (DCFDA) using flow cytometry. (**C**) Effect of NAC on ROS production in WAM-treated LNCaP cells. Data are expressed as means ± SD. * *p* < 0.05 vs. the untreated control.

To examine the role of ROS in WAM-induced apoptosis, ROS was quantified using a DCFDA cellular ROS detection kit in WAM-treated LNCaP cells using flow cytometric analysis. Here, WAM significantly enhanced ROS production in a dose-dependent fashion in LNCaP cells compared with EAM (Figure 4A,B). Conversely, the ROS scavenger N-acetyl L-cysteine (NAC) reduced the ability of WAM to generate ROS in WAM-treated LNCaP cells (Figure 4C).

### 2.5. WAM Suppressed DHT-Induced AR and PSA and Also Reduced the Protein Stability of AR in LNCaP Cells

**Figure 5:**
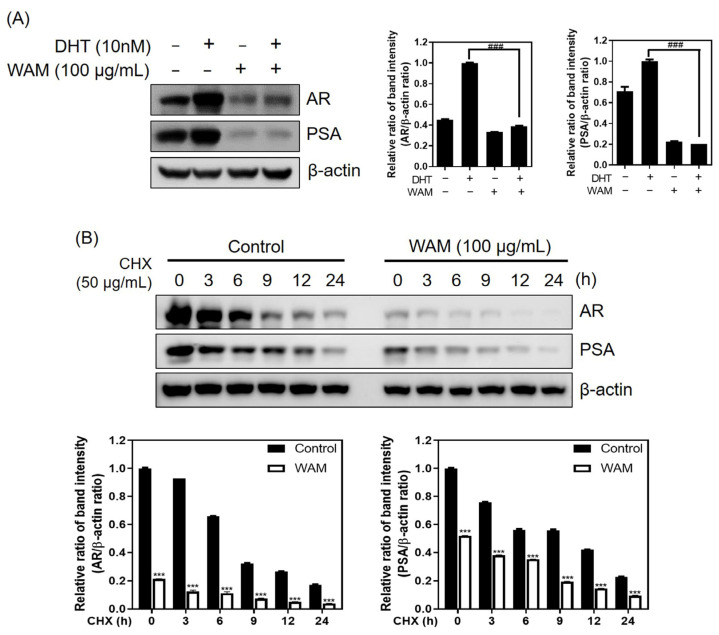
WAM suppressed the protein expression of DHT- and CHX-treated AR and PSA in LNCaP cells. (**A**) Effect of WAM on the AR and PSA in LNCaP cells in the absence or presence of DHT (10 nM) for 24 h. Data are expressed as means ± SD. ### *p* < 0.001 vs. the DHT treated control. (**B**) Effect of WAM on the AR and PSA in LNCaP cells in the absence or presence of cycloheximide. LNCaP cells were subjected to Western blotting in the absence or presence of cycloheximide (50 μg/mL) for 0, 3, 6, 9, 12, and 24 h. Data are expressed as means ± SD. *** *p* < 0.001 vs. untreated control.

To determine whether WAM inhibits DHT-induced AR-related proteins, Western blotting was performed. WAM reduced the expression of DHT-induced AR and PSA in LNCaP cells (Figure 5A). To examine the effect of WAM on protein stability, a cycloheximide assay was performed in LNCaP cells. Here, WAM significantly decreased the stability of the AR and PSA compared to the cycloheximide control in LNCaP cells (Figure 5B).

### 2.6. Association with HSP27 and AR Signaling in Prostate Cancer Cells

**Figure 6:**
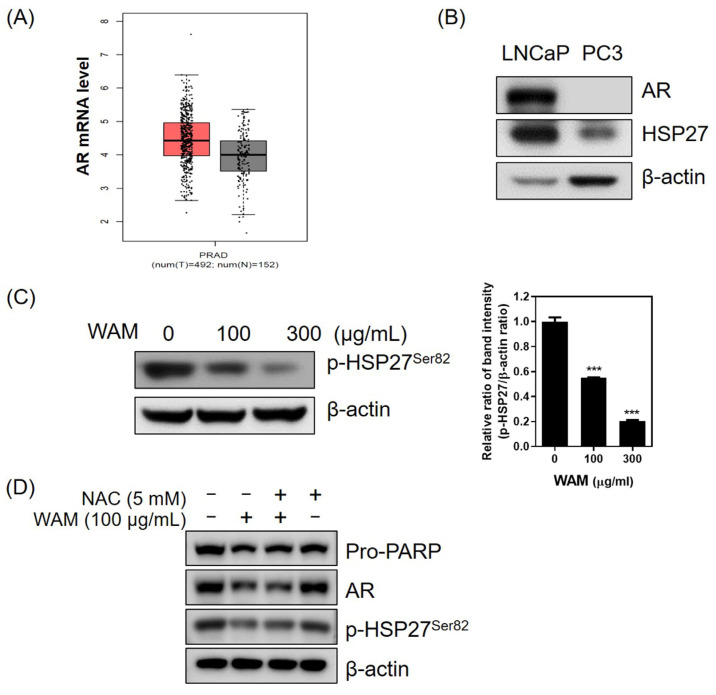
WAM reduced the expression of the AR and HSP27 in LNCaP cells. (**A**) mRNA expression profiles of HSP27 in PRAD tumor samples and normal samples, which were downloaded from the TCGA and GTEx databases. (**B**) Endogenous expression levels of the AR and HSP27 in LNCaP and PC3 prostate cancer cells. (**C**) Effect of WAM on p-HSP27 in LNCaP cells. Cell lysates were prepared and subjected to Western blotting for p-HSP27 (Ser82). (**D**) Effect of NAC on the capacity of WAM to abrogate the expression of pro-PARP, the AR, and p-HSP27 (Ser82) in WAM-treated LNCaP cells. LNCaP cells were pretreated with ROS scavenger NAC (5 mM) for 1 h and treated with WAM (100 μg/mL) for 24 h. The whole-cell lysates were subjected to Western blotting for pro-PARP, the AR, and p-HSP27 (Ser82). Data are expressed as means ± SD. *** *p* < 0.001 vs. untreated control.

The TCGA database revealed that the AR is highly expressed in prostate adenocarcinoma (PRAD) tissues compared with normal samples (Figure 6A). To test the expression of HSP27 and the AR in PC3 (AR-negative) and LNCaP (AR-positive) prostate cancer cells, Western blotting was performed. Here, HSP27 was highly expressed in LNCaP cells, while it was only slightly expressed in PC3 cells (Figure 6B). Consequently, WAM decreased the phosphorylation of HSP27 on Ser82 in LNCaP cells (Figure 6C), while ROS scavenger N-acetyl L-cysteine (NAC) reduced the ability of WAM to decrease the expression of pro-PARP, AR, and p-HSP27^Ser82^ in WAM-treated LNCaP cells (Figure 6D).

### 2.7. WAM Suppressed the Interaction and Colocalization of p-HSP27/AR in LNCaP Cells

**Figure 7:**
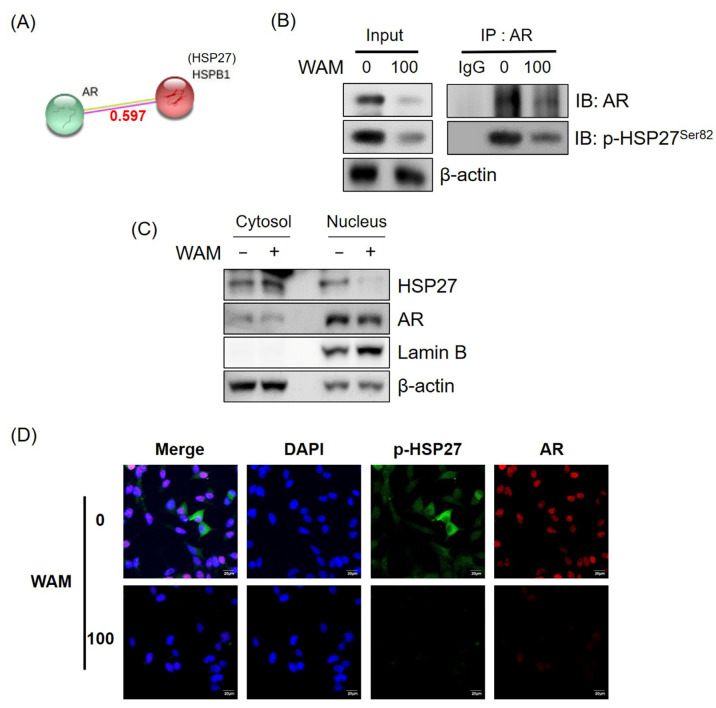
WAM disturbed the interaction between HSP27 and the AR in LNCaP cells. (**A**) Protein–protein interaction score between the AR and HSP27 according to the STRING database. (**B**) Effect of WAM on the interaction between HSP27 and the AR in LNCaP cells using IP. LNCaP cells were treated with WAM (100 μg/mL) for 24 h and immunoprecipitation was performed in whole-cell lysate with the antibodies of the AR and p-HSP27. (**C**) Effect of WAM on the expression of HSP27 and the AR in the nuclear and cytoplasmic fractions of LNCaP cells. The nuclear and cytoplasmic fractions of LNCaP cells were isolated and subjected to Western blotting with the antibodies of HSP27, the AR, Lamin B, and β-actin. (**D**) Effect of WAM on the colocalization of HSP27 and the AR in LNCaP cells assessed using immunofluorescence. The cells were stained with antibodies, the AR, p-HSP27, Alexa Flour 488 goat anti-rabbit, Alexa Flour 549 goat anti-mouse, and DAPI, and were visualized under a FLUOVIEW FV10i confocal microscope. Scale bar = 20 μm.

To confirm the inhibitory effect of WAM on the interaction and translocation of p-HSP27/AR, immunoprecipitation and immunofluorescence were performed in LNCaP cells treated with WAM. The score of protein–protein interaction between the AR and HSP27 was found to be 0.597 using the String database (Figure 7A). As shown in Figure 7B, WAM suppressed the binding of p-HSP27 with the AR in LNCaP cells. Consistently, the expression levels of HSP27 and the AR were determined in the nuclear and cytosol fractions of LNCaP cells. Herein, WAM suppressed the nuclear translocation of the AR/p-HSP27 (Figure 7C) and reduced the fluorescent expression of p-HSP27 and the AR in LNCaP cells through immunofluorescence (Figure 7D).

## 3. Discussion

In the current study, the apoptotic mechanism of water extract from *Astragalus membranaceus* (WAM) was examined in LNCaP prostate cancer cells in association with ROS-mediated HSP27 and AR signaling. Herein, WAM inhibited the viability of human prostate cancer LNCaP cells and increased the sub-G1 portion, the cleavage of PARP and caspase 3, and the number of TUNEL-positive cells, and inhibited Bcl-2 in LNCaP cells, implying that the cytotoxicity of WAM is mediated by apoptosis in LNCaP cells. Accumulating evidence suggests that androgen receptor (AR)-mediated signaling plays an important role in the development and progression of prostate cancer [20,21]. The AR, a member of the steroid receptor family, is a ligand-dependent transcription factor that mediates androgen and DHT in prostate cancer cells [21,22]. Of note, the AR is associated with cellular chaperones such as HSPs in the cytosol in its inactive state [23]. After binding to androgens, such as testosterone and dihydrotestosterone (DHT), the AR is translocated into the nucleus, binds to androgen response elements, and modulates the expression of AR target genes such as the PSA and TMPRSS2 [3,24,25]. Therefore, AR signaling is regarded as one of the most important targets for prostate cancer treatment [24,25]. Here, WAM suppressed the expression of the AR and PSA and inhibited DHT-induced PSA and AR expression in LNCaP cells, implying that WAM inhibits ligand-dependent AR signaling leading to apoptosis in prostate cancer. Consistently, WAM suppressed the protein stability of the AR and PSA in LNCaP cells compared to the DNA synthesis inhibitor [26] cycloheximide control.

Previous studies demonstrated that heat shock protein 27 (HSP27), a member of the small heat shock protein family, is involved in tumorigenesis, survival, metastasis, and drug resistance [27,28,29]. However, recent studies have revealed that HSP27 is involved in the processes of the translocation, folding, and activation and the transcriptional activity of the AR [1,7,30]. DHT binds to the ligand binding site and promotes the separation of HSPs from the AR [31,32]. In addition, androgens induce the phosphorylation of HSP27 and, in turn, promote the expression, transcriptional activity, and translocation of the AR [1]. Matthias et al. reported that HSP27 is an important component of AR signaling as a therapeutic target in prostate cancer [7,30]. Here, WAM suppressed the phosphorylation of HSP27 and the nuclear translocation of p-HSP27 and the AR and disturbed the binding of HSP27 with the AR in LNCaP cells, indicating that WAM inhibits the phosphorylation of HSP27, leading to the downregulation of the AR via the interrupted binding of p-HSP27 with the AR. In addition, Yaxin et al. reported that HSP27 is involved in AR stability [8].

However, the observed cytotoxicity and PARP cleavage in normal prostate epithelial RWPE-1 cells after 72 h of exposure to WAM is a concerning finding, though WAM has been traditionally used in WAM and chicken soup with safety due to its tonic effect for thousands of years in Asian countries, and also, Szabo [33] reported that cycloastragenol, a bioactive triterpene aglycone from Astragalus root extracts, can be safely used as a modern dietary ingredient. Hence, safety studies on, for example, PK, ADME, and toxicity including the functions of the liver and other organs should be conducted with 300 μg/mL of WAM in animals in the near future.

In summary, WAM increased the cytotoxicity, the sub-G1 population, the cleavages of PARP and caspase3, and the number of TUNEL-positive cells, and decreased the expression of Bcl-2 in LNCaP cells. In addition, WAM increased ROS production; suppressed p-HSP27, the AR, the PSA, and AR stability; inhibited DHT- and CHX-treated AR and PSA expression; and disrupted the interaction of p-HSP27/AR and reduced the nuclear translocation of p-HSP27 and the AR. Conversely, NAC suppressed the ability of WAM to attenuate the expression of PARP, the PSA, and the AR in LNCaP cells. Taken together, these findings demonstrate that WAM induces apoptosis via the ROS-mediated inhibition of HSP27 and the androgen receptor in prostate cancer.

## 4. Materials and Methods

### 4.1. High-Performance Liquid Chromatography Analysis

Based on Park et al.’s paper [34], the profiling of the extract of the dried root of *Astragalus membranaceus* was analyzed using HPLC. In brief, a YMC ODS-AM (4.6 × 250 mm, 5 μm) column was applied at 30 °C with 0.1% formic acid and acetonitrile as the gradient system of the mobile phase under the Water e2695 series HPLC system (Waters Corporation, Milford, MA, USA). The mobile phase was maintained in 5% acetonitrile for 3 min and increased to 20% for 3 min, and then, up to 28% for 25 min. Then, elution was carried out by increasing the mobile phase to 100% acetonitrile for 4 min with a flow rate of 1 mL/min. The absorbance of the UV detector was measured at a wavelength of 254 nm compared to standard chemicals such as calycosin (99%) and calycosin-7-O-β-D-glucoside (99%) with high purity from ChemFaces (Wuhan, China) and Sigma-Aldrich (St. Louis, MO, USA).

### 4.2. Cell Line and Culture

Human prostate cancer cells, LNCaP, PC3, and DU145, purchased from the Korean Cell Line Bank (KCLB, Seoul, Republic of Korea), were maintained in RPMI 1640 with 10% fetal bovine serum (FBS) and 1% antibiotic antimycotic solution (containing penicillin, streptomycin, and amphotericin B) (Welgene, Gyeongsan, Republic of Korea) at 37 °C under a humid environment with 5% CO_2_. Human prostatic epithelial cell line RWPE-1 (CRL-11609) cells purchased from the American Type Culture Collection (ATCC, Manassas, VA, USA) were maintained in Keratinocyte Serum-Free Medium (K-SFM) supplemented with 0.05 mg/mL bovine pituitary extract (BPE), 5 ng/mL human recombinant epidermal growth factor (EGF), and antibiotic–antimycotic (Gibco, Big Cabin, OK, USA).

### 4.3. Cell Viability Assay

The viability of LNCaP, PC3, DU145, and RWPE-1 cells was measured using a 3-(4,5-dimethylthiazol-2-yl)-2,5-diphenyltetrazolium bromide (MTT) assay. The cells (1 × 10^4^ cells/well) were exposed to various concentrations of WAM and ethanol extract from *Astragalus membranaceus* (EAM), incubated with MTT solution (1 mg/mL) for 2 h, and then, treated with dimethyl sulfoxide (DMSO) for 20 min. The optical density (OD) was measured using a microplate reader (Molecular Devices Co., San Jose, CA, USA) at 570 nm. Cell viability was evaluated as a percentage of viable cells in the WAM-treated group versus the untreated control.

### 4.4. Cell Cycle Analysis

LNCaP cells (2 × 10^5^ cells/well) were treated with WAM (0, 100, or 300 μg/mL) for 24 h and fixed in 70% ethanol overnight. The cells were incubated with RNase A (10 mg/mL) for 1 h at 37 °C and stained with propidium iodide (50 μg/mL) for 30 min at room temperature in the dark. The DNA content of the stained cells was analyzed using FACSCalibur (Becton Dickinson, Franklin Lakes, NJ, USA.

### 4.5. Measurement of ROS Generation

To determine the levels of ROS production, 2,7-Dichlorofluorescein diacetate (DCFH-DA) was used. LNCaP cells (2 × 10^5^ cells/well) were treated with WAM (0, 100, or 300 μg/mL) for 24 h, and then, with 20 μM DCFH-DA for 30 min at 37 °C. ROS fluorescence intensity was measured using FACSCalibur (Becton Dickinson, Franklin Lakes, NJ, USA).

### 4.6. Terminal Deoxynucleotidyl Transferase dUTP Nick End Labeling (TUNEL) Assay

DNA fragmentation was analyzed using an In Situ Cell Death Detection kit (Roche Diagnostics, Basel, Switzerland). LNCaP cells (5 × 10^4^ cells/well) were plated onto 4-well chamber slides and exposed to WAM (100 μg/mL) at 37 °C for 24 h. The cells were fixed in 4% paraformaldehyde for 1 h and treated with TUNEL buffer containing fluorescein isothiocyanate fluorescein-12-dUTP for 1 h at 37 °C in the dark. The slides were mounted in a medium containing DAPI (Vector Laboratories, Burlingame, CA, USA) and visualized under a FLUOVIEW FV10i confocal microscope (Olympus Corporation, Tokyo, Japan).

### 4.7. Western Blotting

LNCaP cells were exposed to various concentrations of WAM for 24 h, lysed with lysis solution (50 mM Tris–HCl, pH 7.4, 150 mM NaCl, 1% Triton X-100, 0.1% SDS, 1 mM EDTA, 1 mM Na_3_VO_4_, 1 mM NaF, and 1× protease inhibitor cocktail) containing protease inhibitors (Roche Diagnostics GmbH, Mannheim, Germany), and phosphatase inhibitors (Sigma-Aldrich; Merck KGaA, St. Louis, MO, USA) on ice, and spun down at 14,000× *g* for 20 min at 4 °C. The supernatants were collected and quantified for protein concentration using an RC DC protein assay kit (Bio-Rad, Hercules, CA, USA). The protein samples were separated on 10~12% SDS-PAGE and transferred to 0.45 μm nitrocellulose membranes for detection with antibodies for the AR (BD, Franklin Lakes, NJ, USA), PARP, the PSA, caspase3, cleaved-caspase3, Bcl-2, p-HSP27, HSP27 (Cell Signaling Technology, Beverly, MA, USA), and β-actin (Sigma, St. Louis, MO, USA). The blot images underwent semi-densitometric analysis using ImageJ 1.51k software.

### 4.8. Co-Immunoprecipitation

LNCaP cells were lysed in NP-40 lysis buffer, and then, 300 μg protein extracts were immunoprecipitated with an AR antibody. Thereafter, protein A/G sepharose beads (Santa Cruz Biotechnology, Santa Cruz, CA, USA) were applied. The final precipitated proteins were subjected to immunoblotting with the indicated antibodies of the AR, p-HSP27^ser82^, and β-actin.

### 4.9. Immunofluorescence

LNCaP cells were fixed on a poly-L-lysine-coated slide in 4% paraformaldehyde and permeabilized in 0.1% Triton X-100. The permeabilized cells were then incubated with 3% bovine serum albumin (BSA) in PBS for 1 h, followed by immunostaining with rabbit polyclonal anti-p-HSP27 and mouse monoclonal AR antibodies. Rabbit and mouse IgG FITC antibody H&L (Abcam, Cambridge, MA, USA) was used as the secondary antibody. The immunostained cells were mounted in a medium containing DAPI (Vector Laboratories, Burlingame, CA, USA) and visualized using FLUOVIEW FV10i confocal microscopy (Olympus Corporation, Tokyo, Japan).

### 4.10. Statistical Analysis

For the statistical analysis of the data, GraphPad Prism 5 (GraphPad Software, Inc., La Jolla, CA, USA) was used. All data represent means ± standard deviation (SD). Student’s *t*-test and one-way analysis of variance were used for comparison of control and WAM-treated groups. A statistically significant difference was accepted at *p* < 0.05 between the control and WAM-treated groups.

## Data Availability

The data that support the findings of this study are available from the corresponding author upon reasonable request.
